# Supplementary material for: Impact of a Breathing Intervention on Engagement of Abdominal, Thoracic, and Subclavian Musculature during Exercise, a Randomized Trial
Source: J Clin Med. 2021 Aug 10;10(16):3514. doi: 10.3390/jcm10163514 (PMC8397177; doi:10.3390/jcm10163514)
Supplement: Supplementary file 1 [file jcm-10-03514-s001.zip › jcm-1307116-supplementary file.pdf]

Breathing exercises – first part (the first 4 weeks)

Principle: we always take breath and exhale through a nose

Localized breathing - practice
1. Lie on the back with legs slightly spread to the width of the pelvis, bend the legs, rest the feet on the pad (gently push the heels and whole feet into the pad, thus straightening the lumbar spine), and bring your chin slightly to your chest.
We're focused on our breath. We observe inhalation and exhalation.
a) **Abdominal breathing (lower)**. We place our palms on our stomachs and take our breath into the abdomen. Pressure rises under the hands as the breath is taken; the abdomen arches slightly up and to the sides. With the exhalation, the belly drops and the tension is lost. Repeat 10 times.
b) **Thoracic respiration (moderate)**. We place our palms on the lower ribs. By strengthening the abdominal cavity during exhaliation, we limit abdominal breathing. Then we take the breath into the area of the lower ribs and watch as the ribs stretch to the sides (fingers of the hands are delayed) and with exhalation the ribs coming closer together (fingers of the hands approach). Repeat 10 times.
c) **Subclavicular respiration (upper)**. We put our hands on the upper ribs under the clavicle. We lead our breath to the upper parts of the lungs, we see that when we breathe, mainly the upper ribs and sprouts rise (the chest opens and rises slightly without lifting the shoulders), the abdomen and lower ribs almost do not participate in this movement (as with chest breathing, we strengthen the abdominal muscles during exhalation and thus limit abdominal breathing). Repeat 10 times.

2. Sit on your heels (position: Sitting kneel). The heels should be directly under the buttocks (the feet and heels do not part). The head, shoulders and buttocks are in one line, perpendicular to the ground. Hands are loosely placed on the upper thighs with their palms. If this position is not comfortable for the trainers, the exercise can be carried out in the Turkish sit. We focus on the ischial spine and then try to push it slightly into the pad. The head pulls upwards behind the top, the spine is upwards, the shoulders and shoulder blades are pulled back and down towards the pelvis.

a) **Abdominal breathing (lower)**. We place our hands as high as possible on the thighs with the back upwards, and the thumb and index finger are connected by their ends so that they form a ring. The other fingers are loosely stretched and pulled together. Then turn our hands on the thighs, the connected thumb with the index finger goes to the groin. We observe that when inhaled, the abdomen slightly arches forward and to the sides. Whenexhaling, the abdomen drops. We breathe 10-20 times.

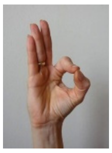

b) **Thoracic respiration (moderate)**. The starting position in the heel seat remains the same. The thumb and for index finger are reassued to form a ring, the other three fingers are curled up in the palm of your hand. The hand is again laid with the palm down on the upper part of the thigh. We observe that both the inhalation and exhalation are realized in the lower part of the chest in the area of the lower ribs. We breathe 10-20 times.

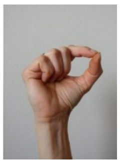

c) **Subclavicular respiration (upper)**. The starting position in the heel seat remains the same. The thumb of the hand is bent into the palm and is covered with other fingers, which are slightly clenched in the fist. The hand is again laid with the palm down on the upper part of the thigh. We observe that both the inhalation and exhalation are realized in the upper part of the chest under the clavicle. We breathe 10-20 times.

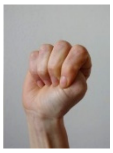

In case of pain, fatigue or cramps in the lower limbs, we go into the pattern of kneeling and loosen the legs, or shake them, and then return to sitting on the heels or the Turkish sit.

Full breath - practice
Basic position: the cat pose, let's check that we have knees under the hips that are far apart in the width of the pelvis, and hands under the shoulders, which are far apart in the width of the shoulders, with our fingers we turn slightly together, the elbows are not stretched
Concentration: on the spine and the perception of full breath (with a breath first led to the abdominal area, through the middle thoracic part the inhalation is directed up to the subclavicular area, <u>the chest opens without raising the shoulders</u>)

- With a breath, we gradually bend the spine from the coccyx, then the head rises into a bend, the viewpoints are upwards
- With exhalation from the coccyx, we slowly hunch the spine; then lower the head, the viewpoints to the navel.

- By pulling the abdomen against the shoulders during exhaling, we support active hunching in the area of the lumbar spine.
- We do the same exercise 10 times to the rhythm of our breath.

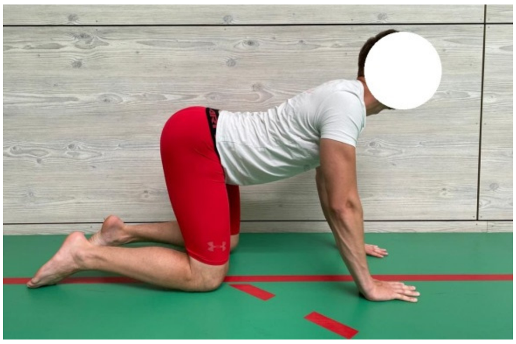

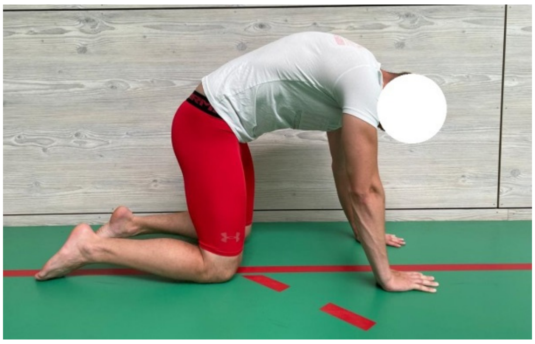

- Then we sit on our heels in the position of sitting on our heels, observe our breath, realize full breath (with a breath first led to the abdominal area, through the middle thoracic part to the subclavicular area)

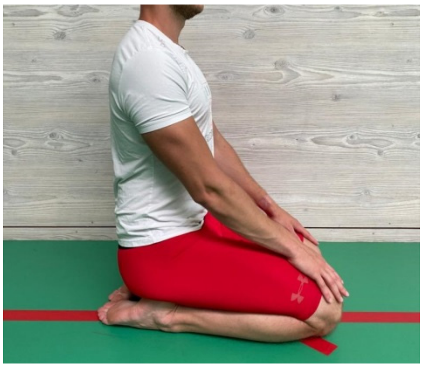

- Then we return to the cat position and then we sit on the heels, leaving our hands in the extension with the forearm laid on the ground, resting the forehead on the mat and relaxing for a while. The position allows to breathe to the back of the lungs, thus a massage of the abdominal organs.

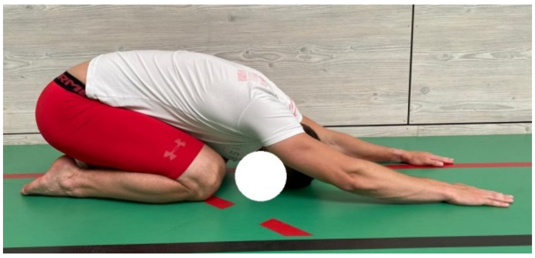

Breathing Exercises Part II – last 4 weeks

Principle: we always take inhale and **exhale through THE NOSE**, breathe relaxed without effort with a slight emphasis on **exhalation,** practice SLOWLY in accordance with the breath, focus on the movement performed

1. Sitting kneel, the heels are directly under the buttocks (feet and heels do not part). The head, shoulders and buttocks are in one line, perpendicular to the ground. Hands are loosely placed on the upper thighs with their palms.

- With a deep breath inhale, we rise our arms and stretch the spine upwards.
- With exhale, we put our hands on the thighs, straighten the spine, head, shoulders and buttocks are in one line, perpendicular to the ground.
- With inhale we stretch the arms forward and at the same time we go high kneel (the transition to the high kneel is slow, and lasts as long as a deep breath, the head is in the extension of the spine perpendicular to the ground, expanded chest).
- With expiration, we gradually move to the cat position (the arms are perpendicular to the ground on the width of the shoulders, as well as the thighs are perpendicular to the ground in the width of the pelvis, the elbows are not stretched, the back and head are in one straight straight), here we stay for a few breaths. It is

rhythmic deep breathing (we can count in our mind for 4times inhalations and 4 times exhalations)(One time is roughly one second).

- We're still counting in our mind, and we're starting to add movements to it. With an inhale (for 4 seconds) we go into a bend, the chest is dilated and with exhalation (for 4 seconds) we go to hunch, the abdomen is pulled against the shoulders. The head is bent between the arms pointing slightly towards the pelvis. Repeat six times.
- With an inhale, we go to the high kneel and at the same time stretch the arms forward.
- With an exhale, we sit on our heels and put our hands on the thighs.
- Then we return to the cat position and sit on the heels, leaving our hands in the extension with the forearm laid on the ground, resting the forehead on the mat and relaxing for a while

2. Sit on your heels (position: Sitting kneel). The heels are directly under the buttocks (feet and heels do not part). The head, shoulders and buttocks are in one line, perpendicular to the ground. Hands on your hips.

- With an exhale, we freely tilt the head, the back remains straight.
- With an inhale, we go to the high kneel, push the pelvis forward, the head goes only into a slight tilt back.
- With exhale, we slowly return to the sit on the heels, the back remains straight, slightly pull the abdomen against the lowerback, lower the head freely on the chest, loosen the hands.
- Repeat 6 times (inhalation is carried out for at least for 4 times, exhalation is longer).

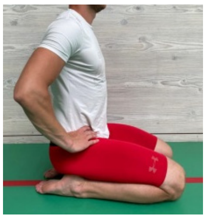
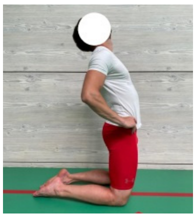

3. Sit on your heels, lean on the hands behind the back by putting the hands on the mat, fingers touching the toes.

- With exhale let the head fall freely to the chest, pull in the abdomen and lighten the hands.
- With an inhale (3-4 times long) we smoothly lift the pelvis as high as possible, the head is in an extension or a slight tilt.
- With exhale lower the pelvis down and return to the starting position.
- Repeat six times.

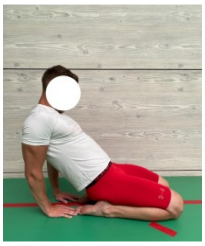
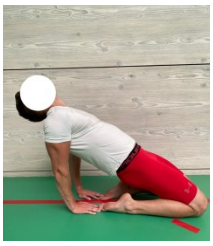

4. Sit on your heels, lean forward and rest our palms on the pad just before the knees (fingers pointing forward). Push the palms into the pad, straighten the back, strengthen the abdominal wall, the head is in the extension of the spine. We breathe deeply into the chest, breathe for 6 times, then lower the forehead on the pad, place the arms loosely around the head and continue to breathe deeply for a while, relax.

5. Sit down and lean behind the back first with one elbow then the other elbow. We transfer weight to the elbows and forearms and tilt the head freely backwards. Our chest is expanded and we're breathing deeply, our legs are relaxed. We breathe six times. Then we loosely lower ourselves to the lie on the back and relax for a while.

6. Sit on your heels.

- With an inhale, put the arms behind the back and intertwine the fingers and stretch the arms..
- With an exhale we bend forward and rest the forehead on the pad (we remain on our heels), and then we begin to raise our hands and finish exhale (we try exhale all air out of the lungs).
- With an inhale, we put our hands back on our back (the head remains resting on the pad).
- In this position, we begin to exhale again, and when we are at the end of the exhaling, we begin to rise the arms again and finish the intense exhale.
- Repeat six times.
- Finally, we slowly rise to sit on our heels, the head rises last.

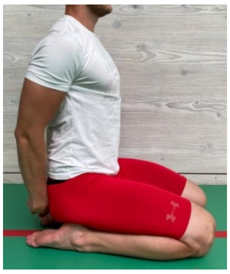
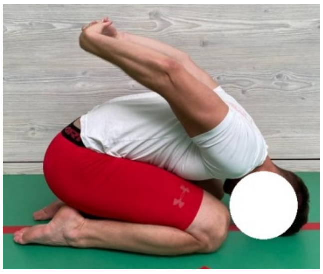

This exercise can also be done standing up; we perform a forward bend and do the exercise described above (a variant for individuals who do not like to sit on their heels).

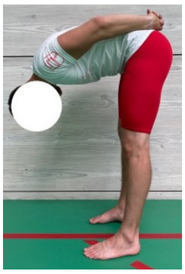 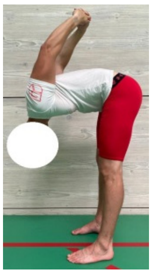

7. Lie on the back with legs slightly spread to the width of the pelvis, bend the legs, rest the feet on the pad (gently push the heels and whole feet into the pad, thus straightening the lumbar spine), we tighten the chin slightly to the chest. Hands by your side.

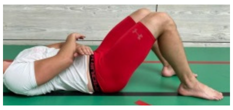
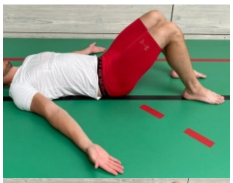
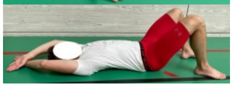

- With exhale, we raise our hands and cross them over the body (above the abdomen), closing the chest to excite as much air as possible.
- With an inhale, we lead our arms on the ground (palms upwards) above our head where we cross the hands again.
- While exhale, we lead the arms on the ground back to the body, and at the end we cross our hands above the abdomen again.
- Repeat six times. The movement of the hands is in harmony with the breath. We perceive the activity of the chest during the movement of the hands.

8. Sit, stretched legs, palms rest behind your back on the pad, fingers pointing to the buttocks.
- In a posture coupled with a breath, we turn our palms forward and, in a smooth arc across the footwork, brace smoothly, crossing our forearms over our heads and lowering our heads into a slight tilt.
- With exhale, we lower the pelvis back into the seat, bend our legs and grab the knees with our hands (right hand right knee and left hand left knee) and pull them to the head and at the same time pull the head to the knees. This will compress the abdomen with thighs and achieve a deep exhalation. At the same time, we're going to stretch our backs.
- With an inhale, we stretch our legs, lean behind our backs on our hands and lift the pan again.
- Repeat six times.

9. Stand with feet together.
- With an inhale, we turn the palms forward and, in a smooth arc rise arms, cross the forearm above the head and lower the head into a slight bend.
- With an exhale, arms will smoothly go back down and behind the back will fingers intertwine.
- We continue to exhale further into the deep bend forward; our hands are loosely placed on our backs. To complete the exhalation we stretch arms and turn the palms out.
- With an inhale turn the palms back and put the arms loosely on the back, then slowly return to the standing position. When we reach an upright stand, unplut our hands, turn the palms forward and in a smooth arc rise the arms and cross the forearm above the head and lower the head into a slight bend.
- Repeat six times.

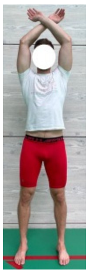 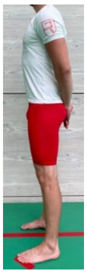 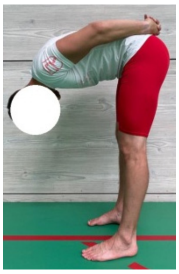 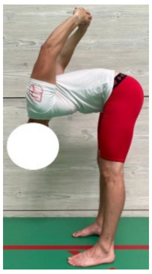

10. Lie on your back.
Variant A. stretching one side of the body:

- With an inhale rise the right arm over the head and stretch it away from the body, while pulling the right foot from the heel into the distance. When exhaling put the arm back next to the body.
- During exercise, we check the lumbar spine to avoid bending.
- We'll do the same exercise with our left hand.

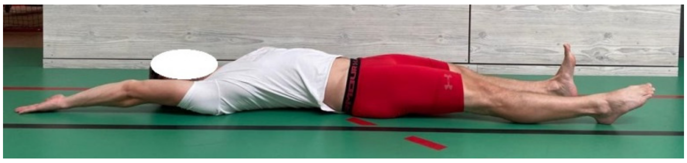

- **Variant B.** stretching in the diagonal of the body:
- With an inhale rise the right arm over the head and stretch it away from the body, while pulling the left foot from the heel into the distance. When exhaling put the arm back next to the body.
- During exercise, we check the lumbar spine to avoid bending. We'll do the same exercise with the left hand and right foot.

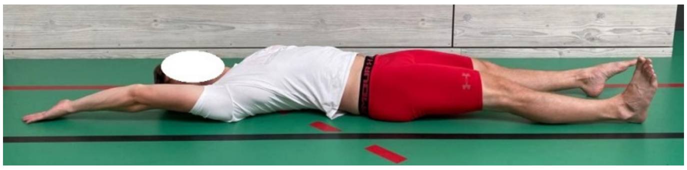

- **Variant C.** stretching both sides of the body at the same time:
- With an inhale rise the arms over the head and stretch them away from the body, while pulling both foot from the heel into the distance. When exhaling put the arm back next to the body.

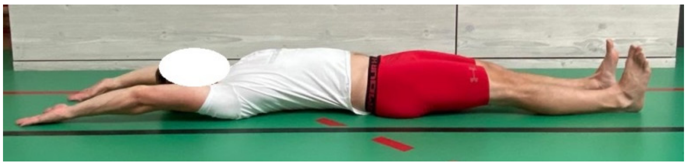

During exercise, we check the lumbar spine to avoid bending.

- **At the end, we lie relaxed on our back with our legs slightly spread to the width of the pelvis, stretching out into the distance.** We place the arms loosely along the body with the palms on the pad, then push the palms slightly into the pad, and at the same time stretch towards the heels, then loosen and turn the palms upwards. Pull the chin slightly to the chest. Close your eyes and watch the breath. Relax.
